# Book Reviews

**Published:** 1815-07

**Authors:** 


### CRITICAL ANALYSIS
### OF RECENT PUBLICATIONS
IN THE
### DIFFERENT BRANCHES OF PHYSIC, SURGERY, AND MEDICAL PHILOSOPHY.

---

### *Philosophical Transactions of the Royal Society of London for the Year* 1814. Part II.

### *Observations on the Functions of the Brain.* By Sir Everard Home, Bart. F.R.S.

THIS truly valuable paper contains a short history of cases at different times under Sir Everard Home's cognizance, in which the brain has been injured or diseased. We have long expressed our wish for such registers, and are glad to meet with the present from a public character, and in a compilation which will be attended to in every part of the civilized world. We shall offer the facts, as they occur, in the author's words; and, as the work is divided into sections, shall venture a few hints and remarks on each.

"I. *Effects produced by an undue pressure of water upon the brain.*

"Before I enter," says Sir Everard, "into the particular effects that take place when pressure is made upon the brain by means of water, it is necessary to mention that sudden pressure of any kind upon the cerebrum, takes away all sensibility, whether made upon the external surface through the medium of the dura mater, or upon the internal parts through the medium of the ventricles, and sensibility returns as soon as the unusual pressure is removed.

"Faintness is the consequence of the pressure, to which the cerebrum has been accustomed, being suddenly taken off.

"I am induced to believe that pressure to a certain degree uniformly kept up, is necessary for the performance of the healthy functions of the cerebrum; and any increase or diminution of this pressure puts a stop to them. It is asserted, that, in addition to this pressure, the pulsatory motion of the blood in the arteries of the cerebrum is also necessary; but the late John Hunter, whose accuracy in a point of this kind is not to be doubted, retained his senses, although the heart had apparently ceased to act.

"Although insensibility is the common effect of undue pressure upon the cerebrum, it appears, from what will be stated, that it is not a necessary consequence of undue pressure upon the cerebellum.

"The facts which have been stated appear to point out the use of the water in the ventricles of the brain, and they account for the the great variety which is met with in the form and extent of the posterior cornua of the lateral ventricles, their size varying according to the quantity of water which is necessary to keep up the pressure required.

"The size of the ventricles would appear to be very immaterial, since even when they are increased so as to contain above six ale pints, the functions of the brain are all carried on, and the growth of the body proceeds; but, after the skull is completely ossified, an increase of two or three ounces produces insensibility.

"That the ventricles should admit of being increased to so great an extent, without any of the senses or faculties of the brain being destroyed, is in itself a curious fact, and of so much importance with respect to the physiology of the brain, that I shall detail the two following cases, which illustrate one another.

"In the one, the accumulation of water proceeded, as it will appear, as far as it could go without materially impairing the organ; it then stopped, and the boy grew up with all his faculties: in the other, the water continued to increase, the substance of the cerebrum was absorbed, and the faculties of the brain were destroyed.

"A boy, at a month old, had so rapid an increase of the size of his head, as to evince an accumulation of water in the brain; and, when he was five years old, the head was so large that the parents, judging from recollection, believe that it never after increased. It was so transparent, that when exposed to the sun the rays passed through it as they would through a horn lantern. He was unable to walk. At this age, he caught the natural small-pox, which was so violent as nearly to prove fatal. Upon his recovery, the head showed no disposition to increase, and the child in all respects began to improve, and for the first time learnt to walk. At fourteen, the skull appeared completely ossified. At nineteen years, the time I saw him, he was five feet six inches high; his head measured in circumference 33½ inches. He had grown in the course of the last year about two inches, which is more than he had usually done in any one year.

"All the organs of sense are entire; savoury food is agreeable to his taste, but he is moderate in eating. His sight is good, but looking with attention at objects more than half an hour appears to strain his eyes. His head is so heavy, that the muscles of the neck are unable to support it for many hours together: when he lies down, the head is supported by another person.

"He sleeps with most ease on the right side, and the left side of the head appears to the eye to be rather the largest. In lying down, there is what he describes to be a momentary thrilling heat felt on the upper part of the brain, in the line of the longitudinal sinus. Lying on his back strains his eyes so much, that he cannot continue in that posture; stooping forwards brings an oppression upon his eyes. The least weight in his hand, as a tea-cup, makes it tremble: all sudden noises jar his head, and produce giddiness. When he falls down, the jar renders him insensible; at one time this this was the case for fifteen minutes, without being attended with any bad consequences. His head aches when exposed to heat. He has had no illness since the small-pox. His sleep is easily broken: he never dreams. He is fond of reading and writing; has a taste for poetry, and can repeat verses out of Cowper. His memory of common things is very good. He never expressed any attachment or passion for women. He is of a mild disposition, but, when irritated, his whole frame is in a state of agitation, which, however, soon goes off.

"In another boy, the enlargement of the head was perceived at three months, and increased for three years, and then appeared to be stationary; and the child till that period was sensible. The upper part of the skull from that time began to ossify, and in three years more there was only an irregular space at the fontanelle, and a small space between the two portions of the os frontis remaining open. The child continued sensible till three years old, and then became gradually less so; did not know what it did; heard sounds, but could not see. At six years old he died.

"The child was three feet three inches high, the skull twenty-seven inches round; the water contained in the two lateral, and third, ventricles, was six ale pints and a half in quantity. The cerebrum formed a thin case of medullary substance surrounding this cavity. The cerebellum was entire.

| | lb. | oz. | dr. |
|---|---|---|---|
| The weight of the whole brain was .................. | 2 | 3 | 1 |
| The weight of the whole brain of a child between six and seven years ............................ | 2 | 12 | 0 |

The preceding facts explain satisfactorily that the cerebrum is made up of thin convolutions of medullary and cortical substance surrounding the two lateral ventricles; which are unfolded when the cavities of those ventricles are enlarged, and in this unfolded state the functions belonging to this part of the organ can be carried on.

"Although the quantity of water may be so much increased without material injury to the functions of the brain, when the skull is not ossified, yet after that period even a few ounces in the lateral ventricles have been known to produce so much undue pressure, as to bring on head-ache, general uneasiness, a sensation as if the head were too large, loss of spirits, convulsions, loss of memory of recent events, idiotism, insensibility, and death.*

"When

"* One ounce and half of water in the lateral ventricles, at 4½ years old, were accompanied by pain in the head, and great general irritability. Five ounces, at 7 years, by insensibility for fourteen days before death. Two ounces and one half, at 9 years, by violent head-aches, an unwillingness to be moved, and stupor. Three ounces, at 11 years, were attended with loss of recollection of

"When the water, instead of being contained in the general cavities of the lateral ventricles, is principally confined to their posterior and anterior cornua, the effects sometimes are occasional constipation, pain in the bowels, and lower part of the belly.* When accumulated in the third ventricle, without any increase in the lateral ones, distressing pain in the head, loss of speech, and insensibility have occurred. † When accumulated in the ventricles of the brain, and also under the tuberculum annulare, painful sensations in the stomach, bowels, lower belly, and across the legs, have been met with. ‡ When not only in the ventricles, but between the tunica arachnoides and pia mater over the hemispheres, and also upon the tubercula quadrigemina, the apparent consequences in one case were depression of spirits, pain in the back of the head, and mania.§ When in the ventricles, and also between the tunica arachnoides and pia mater, and between the dura and pia mater, melancholy, imbecility, apoplexy, and paralysis of one

---

of recent events. This began at the age of 10. A stupor of twelve days preceded death. Four ounces, at 11 years, were followed by the sensation of the head being too big, loss of speech, insensibility, convulsions. Eight ounces, at 74 years, were accompanied by a state of idiotism for ten days, and then death.

"* Five ounces of water, contained principally in four small cells, two in the anterior and two in the posterior cornua of the lateral ventricles, at 6 years of age, were the only evident cause of pain in the lower part of the belly, occasional constipation, and violent pain in the bowels.

"† Two ounces of water in the third ventricle, enlarged by the layers of the septum lucidum being separated, at 30 years of age, appeared to produce distressing pain in the head, loss of speech, and insensibility. Two drams of water in the third ventricle of an aged dog, were attended for four years by fits like apoplexy, pain in the head only relieved by opium, convulsions, and death.

"‡ Two ounces of water in the lateral ventricles, 1½ ounce under the tuberculum annulare, between the tunica arachnoides and pia mater, at 5 years of age, were followed by painful sensations in the lower belly, stomach, and bowels.

"Two ounces of water in the ventricles, one under the tuberculum annulare, at three years, appeared to produce not only painful sensations in the lower belly, stomach, and bowels, but pain in the head and pain across the legs, as if they were cut with an instrument.

"§ Water in the third ventricle, in sufficient quantity to separate and keep apart the thalami nervorum opticorum, also between the tunica arachnoides and pia mater, over the hemispheres, and under the tubercula quadrigemina, in an adult, were attended by pain in the back of the head, low spirits, and, after drinking wine, very high spirits. This ended in mania, and, after three months, in death.

side, side, have been the accompanying symptoms.* When in the ventricles, where there has been an unusual vascularity of the dura mater, violent affections of the præcordia have occurred in the night during sleep, which have led to suicide.† When between the dura and pia mater in considerable quantity, a state of melancholy and imbecility of mind has been met with.‡"

On this subject we could wish larger illustrations, to prove the necessity of pressure on the brain. The probability is, however, great, and it must be admitted that the proof is difficult. We should wish to have been referred also to the authors who conceive the pulsatory motion necessary.

If it is true that pressure on the cerebellum does not produce insensibility, does not this confirm the modern opinion concerning the confined office of that hitherto supposed most important organ? We have not discovered the statement which confirms Sir Everard's proofs, but the opinion is perfectly consistent with Spurzheim's, who gives only *amativeness* to the cerebellum.

Whether the facts above stated really point out the use of water in the ventricles, we shall not undertake to determine, nor are we quite satisfied that the quantity of water contained in these cavities is of no importance, though we are ready to admit that it must be of less than was at one time believed. This is very well accounted for by the remark that *the brain is made up of convolutions, which are unfolded when*

---

"* Two ounces in the lateral ventricles, one between the dura and pia mater; the space between the tunica arachnoides and pia mater loaded with it, the consequences of attacks of inflammation upon the coverings of the brain in an adult were attended by these symptoms. The paralysis took place after the last apoplectic fit, and death ensued.

"† Two ounces in the lateral ventricles, and a very unusually vascular state of the dura mater, in an adult, was followed by these effects.

"‡ A gentleman fell from a horse. From that time he had head-aches, gradually became melancholy and imbecile. He lived three years in that state. Four ounces of water were found between the dura and pia mater, on the right side. There was an exostosis ⅛th of an inch long from the parietal bone with a sharp point, in contact with that part of the dura mater under which the water lay. There were four ounces in the lateral ventricles, the posterior cornua were very small; there were also three ounces on the basis of the skull."

All these records are valuable, but it is to be regretted that the histories during life are so short, and particularly that, excepting in one instance, we have no account whether there were symptoms of inflammation.—Ed.

*the*

*the cavities are enlarged; and that in this unfolded state the functions belonging to this part of the organ can be carried on.* We know not whether this passage is to be considered as a confirmation of some of Dr. Spurzheim's discoveries, or as a thing well known before his time. The following are Spurzheim's words:

"Let us now examine what change the brain undergoes in the dropsy of the cerebral cavities? It was by many anatomists admitted that, in the hydrocephalus of the cavities, the brain was distended like a bladder; but it was unknown in what this distension consisted; and it seemed inconceivable how a medullary substance could be distended to such thinness without breaking. Walter, at Berlin, Ackermann, at Heidelberg, and many others, admit the existence of the cerebral mass in hydrocephalic persons; but they maintain that this mass is destroyed or disorganised. We maintain that the cerebral substance is not disorganized. We prove our assertion by anatomy and physiology.

"It may be proved by anatomy, that the fibres of the brain are directed vertically or perpendicularly above the cerebral cavities, and that every convolution consists of two layers applied one against another, and which may be separated one from another. Thus, if a great quantity of water be accumulated in the cerebral cavities, and act against the convolutions placed round about the cavities, it separates gradually the two layers whose natural position is vertical, till at last their situation is horizontal. In this manner, in large hydrocephalic skulls, the convolutions are entirely unfolded, and present a smooth surface and a membranous expansion."—*Spurzheim's System*, page 178-9.

"II. *Effects produced by concussion of the brain.*

"Concussion of the brain produces delirium and coma: these symptoms go off; they sometimes in a few days return, and prove fatal.*

"In the torpid state commonly attendant upon any violent shake being given to the brain, the senses are so much impaired that little information can be gained respecting the effects produced upon the internal organs. The bowels have been found, under such circumstances, to be acted on by aperient medicines with great difficulty.†

---

"* This happened in two cases, and no appearances of alteration of structure in the brain were met with after death.

"† A gentleman fell from his horse, and had a concussion of the brain. While in this state, it required 60 grains of jalap and 20 of calomel to procure one evacuation from the bowels."

Perhaps the improved mode of dissecting the brain may enable us to discover changes which take place after such accidents, and which have been hitherto overlooked.—Ed.

"III. *Effects*

"III. *Effects produced when the Blood Vessels of the Brain are preternaturally dilated or diseased.*

"Sudden dilatation of the blood-vessels of the cerebrum, in consequence of exposure to the sun, is sometimes accompanied by delirium; loss of speech and the power of swallowing.

"A dilated state of the veins of the cerebrum has been attended with head-aches, which are very severe when the body is placed in a horizontal posture.

"When the smaller arteries of the cerebrum are preternaturally enlarged while those of the cerebellum are not, delirium has taken place, followed by a fit resembling apoplexy, and a paralytic affection of one side.

"An obstruction to the passage of the blood through the right internal carotid artery was attended by a succession of slight apoplectic fits, unaccompanied by any paralytic affection.

"An aneurismal enlargement of both the internal carotid arteries to the size of marbles projecting into the cavernous sinuses, was the only apparent cause of attacks of mania, with consciousness of being insane."

Several notes are added of cases confirming the above. In some, all the symptoms appear to have been the effect of inflammation. We again regret the studied brevity with which they are related. That the changes which appeared produced death, is only presumed by the history of the dissection, and as no other cause of death is assigned.

"IV. *Effects produced by extravasated Blood.*

"Blood in the lateral and third ventricles was attended by repeated fits of vomiting and coma. In the fourth ventricle, a fit, which in twenty-four hours terminated in death; under the anterior lobes of the brain by hiccoughs and stupor; under the cerebellum, by convulsions of the neck and body, with drawing up of the feet without stupor; in the folds of the pia mater covering one hemisphere, by a paralytic affection of the opposite side, without any other symptom.

"Blood in the folds of the pia mater over the posterior lobes of the brain, and serum in the cornua of both the lateral ventricles, were attended by giddiness, paralysis, straight objects appearing crooked, loss of memory, and at last idiotism. In the right thalamus nervi optici,* extending into the lateral ventricles, by paralysis of the left side of the body, both eye-lids closed, the mouth drawn on one side, a perception of light with the right eye, but not with the left, succeeded by coma. Between the dura mater and skull covering the right hemisphere, by stupor which went off on its removal; but taking off the pressure produced faintness for a few minutes.

"Coagulable lymph spread over the union of the optic nerves,

* This is an odd expression, but, as it is intelligible, we are not disposed to make any objection to it.

the pineal gland, and tuberculum annulare, was followed by permanent contraction of the muscles between the occiput and vertebræ of the neck, dilatation of the pupils, and a great degree of deafness. Serum under the cerebellum by restlessness, convulsion, incessant talking, at times incoherent, and the eyes became insensible to light."

All these positions are illustrated by notes, each relating a dissection in a very few words. By the last it would appear, that pressure on the cerebellum, though not attended with insensibility, produced important effects. But, as two ounces of *serum* were found under the cerebellum, it is probable that the whole was the effect of effusion from inflammation. Hence, by sympathy with the neighbouring parts, the more violent symptoms might be induced, and, after the effusion, the eyes become insensible to light, by pressure extending to the optic nerve.

"VI. *Effects produced from Depression, and by thickening of different Portions of the Skull.*

"Unusual pressure of the skull upon the middle lobe of the brain, was attended with pain in the stomach, torpor of the bowels, nausea, retching, pain between the shoulders, and in the feet. On the upper part of the hemisphere, want of sleep, head-ache, and stupor. Those effects went off upon its removal. On both of the anterior lobes of the brain, heaviness, loss of memory, depression of spirits, bordering on idiotism. On the anterior lobes of the brain, accompanied with water between the tunica arachnoides, and pia mater covering the superior part of the hemispheres, an apoplectic fit, heaviness, loss of memory, and a second apoplectic fit, which terminated in death. On the lower and lateral part of the left posterior lobe of the brain uneasiness in the skin of the left cheek, extending along the chin, throat, and trachea, hissing noise in the ears, inability to speak the words the person wished to articulate, using others in their place, although conscious of doing so, and unable to correct it. Numbness in the arms and legs. These effects ceased on taking off the pressure. On the anterior lobes of the brain, both anteriorly and laterally, with thickening of the pia mater, spasms in the lower extremities, and total loss of memory, so that the person did not know what he had done a few hours before: although in other respects in health. On the lower and lateral portions of the anterior and middle lobes of the brain, head-aches, general wasting, irregularity in the action of the bowels; the feel of inability to swallow, and great distress in the act of swallowing, with great general irritability."

By the notes it appears, that all these cases, excepting the two which were relieved, arose from exostosis, or ossification of a membrane: probably the effect of chronic inflammation, in some instances, perhaps, suddenly assuming an acute form; in others, destroying the patient by long continued hectic. This is, however, only an inference of our own.

Effect

"VII. *Effect of Pressure from Tumours.*

"An hydatid imbedded in the substance of the right hemisphere of the brain, was attended with violent head-aches, and occasional fits similar to those of apoplexy. A tumour in the substance of the posterior lobe of the brain, was attended with derangement of the functions of the stomach and bowels, double vision, and afterwards loss of sight. A tumour pressing on the left hemisphere; settled melancholy, drowsiness after dinner, requiring being carried into the air, which took it off, but it returned on coming back to the table. A tumour in the fourth ventricle, epileptic fits, soreness in the throat, and great pain in the act of deglutition. A tumour in the tuberculum annulare and water in the ventricles, pain in the head, stumbling in walking, the mouth drawn on one side, loss of sight of one eye, although the pupils were not affected; dullness in hearing, difficulty of swallowing, so as to die starved, with all the mental faculties entire."

By the notes, all the tumours were of the incysted kind, filled with substances of different degrees of consistency.

"VIII. *Effects of Injury to the Substance of the Brain.*

"A deep wound into the right anterior lobe of the brain, attended with inflammation and suppuration, produced no sensation whatever; the senses remained entire, and the person did not know that the head was injured.*

"The brain shooting out in the form of fungus, after the dura mater is wounded, has no effect upon any of the nerves, nor is it attended with sensation;† but the inflamed pia mater gives great pain.

"Loss of a portion of the medullary substance of the anterior lobe of the cerebrum, produced no symptoms.‡ Loss of a portion of one of the hemispheres was attended with difficulty of swallowing for twenty-four hours, and slight delirium of short duration.§ Ulceration of the anterior lobe of the brain, as low

---

* From an explosion of gun-powder, a piece of copper three inches long was forced through the eye into the brain; but the arm being also wounded, the person never knew the eye to be injured. When suppuration came on he became insensible, and in a few hours died.

† A fungus from the brain shot out through the dura mater after the operation of the trepan, upon the left parietal bone. The pia mater surrounding it was so sensible, that, when touched, the pain was excruciating.

‡ A boy, five years of age, fractured his skull, and wounded the brain, half an ounce came away. When he grew up he was more acute, and had a better memory than his brother.

§ A fracture of the parietal bone, and wound of the dura mater, produced the injury to the brain.

as the anterior cornu of the lateral ventricle, but not communicating with it, paralysis of both arms.*

"In a case of a penetrating wound into the right hemisphere of the brain with bone forced into its substance, while there was an opening for the discharge of matter, no effects were produced, except when the circulation was much increased, and then only head-ache and numbness in the left side."†

We need not dwell on the value of the notes, which would have been increased, could they have been so far lengthened as to inform us of the future history and character of all the patients who survived.

### "IX. *Effects of Alteration in Structure of the Brain.*

"In a case in which the tuberculum annulare had undergone a change in its texture, and become so hard as with difficulty to be cut with a knife, a considerable quantity of earthy particles being intermixed with the medullary substance of the crura, and other parts of the cerebellum, and the cerebrum and upper part of the cerebellum unusually soft; the effects were, the boy had been an idiot from his birth, never walked, spoke, or understood what was said. Went often three days without food. At sixteen, when he died, was no bigger than a child three years old, except the head, which was as large as it is usually at twelve."‡

### "X. *Effects of Injury to the Medulla Spinalis.*

"Pressure upon the medulla spinalis in the neck by coagulated blood produced paralytic affections of the arms and legs, all the functions of the internal organs were carried on for thirty-five days, but the urine and stools passed involuntarily.§

"Blood extravasated in the central part of the medulla in the neck,

---

* The ulceration took place in consequence of the trepan wounding the dura and pia mater.

† A musket ball fractured and depressed a portion of the superior and posterior part of the parietal bone, but did not penetrate: an abscess formed in the substance of the brain in which the broken portions of bone were lodged. In this state the person was capable of doing his duty as a naval officer from China to England, but died in consequence of an attempt to extract the depressed bone.

‡ The cranium was not completely ossified, the fontanelle being still of a large size. This case was examined by Mr. Brodie.

§ A coagulum of blood, the thickness of a crown piece, was found lying upon the external surface of the dura-matral covering of the medulla spinalis, extending from the fourth vertebra colli, to the second vertebra dorsi. The medulla spinalis itself was uninjured.

neck, was attended with paralytic affection of the legs, but not of the arms.*

"In a case where the substance of the medulla was lacerated in the neck, there was paralysis in all the parts below the laceration; the lining of the œsophagus was so sensible, that solids could not be swallowed, on account of the pain they occasioned.†

"Where the medulla in the back was completely divided, there was momentary loss of sight, loss of memory for fifteen minutes, and permanent insensibility in all the lower parts of the body. The skin above the division of the spinal marrow perspired, that below did not. The wounded spinal marrow appeared to be extremely sensible."‡

Perhaps the improved mode of dissecting the brain may enable us to discover changes which took place after such accidents, and which have hitherto been overlooked.

On a review of this truly valuable article, it is impossible not to be struck with the weight of argument which it every where affords in favour of Dr. Spurzheim's opinion concerning the appropriation of different functions or offices. If they should not always correspond, we must recollect, that the histories taken, as they appear, from a case-book refer to events, some of them imperfectly known, and all of them examined and recorded before the new system was broached, and probably before the improved mode of dissecting the brain was sanctioned by the French anatomists. If we have marked a few instances in which we conceive the relations might have been improved, we hope this will be considered, like the regret always expressed at seeing a generally well-finished performance, which few can imitate, but in which the artist is the first to discover parts he should improve, were he to attempt the same work again. Besides the omissions already noticed, we are anxious to mention another, too common in the history of cerebral dissection—the degree of firmness in the substance of the brain itself. A greater degree of elasticity, with effusions into the cavities, is often the only mark we can discover where we know from the symptoms during life, that inflammation was the cause of death. We were pleased to find the comparative fullness of

* The sixth and seventh vertebræ colli were dislocated; the medulla spinalis externally was uninjured; but, in the centre of its substance, just at that part, there was a coagulum of blood nearly two inches in length.

† The seventh vertebra colli was fractured, and the medulla spinalis passing through it was lacerated and compressed.

‡ The spinal marrow within the canal of the sixth vertebra dorsi was completely destroyed by a musket ball. The person lived four days.

the the vessels on the pia mater so little noticed, as nothing can be more fallacious than such an appearance. We shall now notice another paper by the same author, contained in this number.

*On the Influence of the Nerves upon the Action of the Arteries.* By Sir Everard Home, Bart. F.R.S.

"That the pulsations of the arteries correspond in their frequency with the contractions of the left ventricle of the heart, is universally admitted; and those pulsations continuing in the arteries after the limb to which they belong is rendered paralytic, has led to the belief, that all arterial action is independent of nervous influence.

"The object of the present Paper is to shew that the nerves which accompany the arteries regulate their actions, and it is through their agency that the blood is distributed in different proportions to the different parts of the body.

"The facts which have led me to conclude that this office is performed by the nerves, I shall lay before the Society in the order in which they occurred.

"An officer, who had been wounded by a musket ball in the leg immediately below the knee, came under my care. With a view to find the ball, a seton was passed in the course it had taken, and brought through the skin just beyond the part in which it was lodged; a caustic was then applied to the skin just below the tuberosity of the tibia, to which the ligament of the patella is attached: it produced great pain all round the joint and through the leg, and what was very remarkable, the matter in the canal surrounding the seton rose and fell with great violence; this circumstance induced me to feel the pulse at the wrist, and to keep my finger upon it during the time this effect upon the pus continued, which was several minutes, corresponding with the pulse at the wrist, which, however, had been in no degree disturbed.

"The increased action of the arteries, rendered evident by the effect upon the pus, following so closely upon an irritation on the neighbouring nerves, made it clear that it arose from that cause, and brought to my recollection an instance, in which the aorta had been seen pulsating with great violence, in consequence of an irritation upon the nerves of the urinary bladder.

"To ascertain whether such a connection between the actions of nerves and arteries could be demonstrated in a state of health, as well as disease, I instituted the following experiments, which were made in the presence of Mr. Brodie and other gentlemen competent to form a judgment respecting them.

"The carotid artery of a dog was laid bare, the intercostal nerve and par vagum, which form one bundle, were separated from it by a flattened probe for one-tenth of an inch in length, the head and neck were then placed in an easy position, and the pulsations attended to by all present, for two minutes, that the eye might be accustomed to them in their natural state: the nerve passing passing over the probe was then slightly touched with the kali purum. In a minute and a half the pulsations of the exposed artery became more distinct; in two minutes the beats were stronger, and in three more violent; in four minutes the violence was lessened, and in five minutes the action was restored to its natural state.

"This experiment was repeated with the same results upon a rabbit, and in that animal the par vagum was separated from the intercostal nerve, which cannot be regularly done in the dog; and it was found when the par vagum alone was irritated, no change took place in the action of the artery. The carotid artery was chosen as the only one in the body of sufficient size that can be readily exposed, to which the nervous branches supplying it can readily be traced from their trunk.

"This experiment was repeated three different times, so as to leave no doubt respecting the result.

"Having ascertained by these experiments that the increase and diminution of the action of an artery does not depend upon irritability, but nervous influence, I made the following experiments, to determine whether heat or cold had the greatest effect in stimulating the nerves to action.

"The wrist of one arm was surrounded by bladders filled with ice, and after having remained in that state five minutes, the pulse was felt at the same time in that and in the opposite wrist, and the beats were found evidently strongest in the cooled wrist. This experiment was then made with water heated to 120° or 130°, beyond which the heat could not be submitted to, and the pulse was found to be softer and weaker than that in the other arm.

"When one wrist was cooled and the other heated, the stroke of the pulse in the cooled arm had great force beyond that in the heated one.

"These experiments were repeated upon several young men at different ages, and on several women, with an uniform result, and explain the glow produced by the cold bath, and the other beneficial effects of cold bathing, in a more satisfactory manner than has been hitherto done.

"This influence of the nerves upon the arteries, throws considerable light upon some of the most important actions in the animal economy. By its means the same arteries, at different times, allow very different proportions of blood to pass through them, and those employed in furnishing blood for the secretions have the supplies regulated, which explains the use of the system of nerves with which the blood vessels of the viscera are so abundantly furnished.

"The erection of the penis, produced by a particular state of mind, is one of the effects of this influence of the nerves upon the arteries, and the stoppage of the secretions from the same cause is another of an opposite kind.

"The ready supply of blood to a limb by the small anastomosing branches, when the principal arterial trunk is obliterated, depends upon the same cause; and the coagulation of the blood in the arterial trunk leading to a mortified part, arises from the nerves

 having having previously lost their influence over it. On this dominion of the nerves over the actions of arteries depends the growth of the body, the regeneration of parts in those animals in which it occurs, as lizards and others, and the formation of tumours of all the different kinds. The circulation of the blood is therefore no longer to be considered as wholly dependent upon the heart and the elasticity of the arteries, for, although by these alone it can be kept up, the action of the nerves is necessary to regulate the distribution of the blood to the different parts of the body, according as supplies are wanted to carry on the necessary operations of the animal economy."

On this paper we shall offer our remarks with less reserve. That the pulsation of the artery should be stronger under inflammation is not surprising, when we reflect, that inflammation is principally increased arterial action, nor can we wonder if the pulsation should be more palpable, when we know that pressure on an artery always increases its muscular action, as if to overcome a resistance.

The experiments made by Mr. Brodie can hardly be said to demonstrate actions in a state of health, because the operation must induce disease. Nor can we easily understand how the experiments ascertain that the increased or diminished action of an artery does not depend on irritability, unless by irritability is meant that property independent of nervous influence. If this was the object of the experiment, attempts should have been made to stimulate the artery after the nerve was separated from it. We admit, that even these would not have been decisive; but they would have brought us nearer to the proof. We are no better satisfied that the experiments with ice and warm water explain the glow produced by the cold bath in a more satisfactory manner than has hitherto been done. Mr. Hunter had long before remarked, that animals, for the most part, seemed to exist most naturally in a temperature below their own, and in which action must be always excited to generate heat. But surely, how much credit soever we may give to the influence of nerves in regulating arterial action, according to the necessities of a part, it is somewhat bold to assert, that "the coagulation of blood in the arterial trunk leading to a mortified part, arises from the nerves having previously lost their influence over it." If this were really the case, how should blood out of the vessels ever remain uncoagulated? For our parts, when we reflect on the necessity of this coagulation of the blood in the trunk of an artery leading to a mortified part, and that it takes place in arteries considerably nearer the heart than the mortified part,* we should rather

* See Hunter on the blood, p. 23.

impute impute it to the effect, than to the loss, of nervous influence. That the circulation of the blood does not entirely depend on the heart and arteries we are not disposed to question. They are the instruments by which it is performed, as the stomach and intestines are of digestion; but, for the due performance of all these functions, sound health and the regular action of many other parts are necessary.

---

*Edinburgh Medical and Surgical Journal*, No. XLII. for April, 1815.

Art. I.—*On the Malis Dracunculus, or Guinea-Worm;* by C. Chisholm, M. D. F.R.S. &c. &c.

This is a very long, and by many of our readers will be thought a tedious, paper. The generation of intestinal worms is perspicuous enough, but their origin in the human body has been hitherto only conjectured. The guinea-worm is more remarkable as only existing in warm climates, and endemic in certain places and in certain seasons of the year. It is for the most part found in the lower extremities only, and the mode of treatment is now well understood. It becomes us to admit, that the present paper contains much more information than we have met with on the subject of this extraordinary animal; but we cannot, in many places, refrain from suspecting not only some inaccuracy, but a considerable degree of credulity in the author; yet we are satisfied of his good intentions on this as well as on many other occasions in which we have ventured to differ from him.

The guinea-worm, as its name imports, has generally been supposed to be confined to the African coast, but Dr. Chisholm produces many proofs that it is endemic in Grenada, and that it is always confined to those who are in the use of certain descriptions of water. The following particulars he has collected from gentlemen who, unconnected with medicine, could not be warped by any particular theories."

"Mr. Templeman, of the plantations of Edmund Thornton, Esq. thus stated the circumstances relative to the guinea-worm, under his own immediate observation, viz. that the guinea-worm had been epidemic on the plantations under his charge, during a certain period of the year, for seven years, or from 1787 to 1794, when I left Grenada; that the well, of the water of which the field negroes exclusively drink, had been dug nearly about the same time, or about eight years; and was dug in consequence of others more distant from the sea giving an inadequate supply of water;—

water;—that the water has been always perfectly limpid and transparent to the naked eye, and has no disagreeable smell;—that it rises during flood tide about four or five feet, and sinks as much during ebb;—that its taste is brackish, and, although the water itself appear to the eye pure and clear, yet there is always a considerable accumulation of filth at the bottom, notwithstanding its being frequently cleaned out, and this filth smells very disagreeably; that the well is situated about a hundred yards from the sea, near the centre of a small valley running obliquely from the sea into the country;—that it is cut out of a soft tuf;—that the sides and bottom of the little valley are formed of the same substance, having a thin coat of soil, in which cotton and other plants are cultivated or grow spontaneously; that those only who drink of the water of this well have been afflicted with the guinea-worm; the white inhabitants and domestic negroes, who always drink rain-water, have never, except in four instances, had the disease;—that the field-negroes, who drink no other water but that of this well, are universally subject to the worm, so that almost every one of about three hundred has had it, to a greater or less extent, or from one to ten worms at a time in various parts of the body, during the months of November, December, January, and February of every year;—that the annual recurrence of the disease is most regular;—that it first appears generally about the 20th of November, and, before the month of March, when it usually ceases, almost every field-negro on the plantation has suffered by it; that in January it often happens that fifty negroes are laid up at once;—that from the end of March or beginning of April, to the middle of November, not one new instance of the disease has occurred, although it sometimes happens, that the ulcers occasioned by the imperfect extraction of the worm have not entirely healed before the month of June;—that this, however, is very rare. Mr. T. gave some remarkable instances of the disease proceeding from the drinking of the well water. A negro boy, a domestic of his own, had one year, 1793, several guinea-worms; and, as he was supposed never to have drank or made any use of this water, his case was considered as a disproving argument of the efficiency of the water in producing the disease. On a careful investigation, however, it was discovered that, during the summer of 1793, the boy had drank of the well water, but at no time before. Three infants, from five to seven months old, had each a worm in one of their legs. On inquiry of their mothers, it was found, that they had, during the same summer, given them water of the well to drink.

"Mr. Scott, the agent of a large plantation called Grand Ance, similarly circumstanced in almost every respect to the property Mr. Templeman had the charge of, favoured me with answers of precisely the same import. But here a very remarkable proof of the cause of the disease being confined to the internal use of water of wells dug in tuf, and subject to the ebb and flow of the tide, was furnished. Mr. Scott having minutely inquired into the

 nature nature and cause of the worm, which often deprived him of the labour of a large portion of a very numerous gang of negroes, suspected the tuf well-water to be in fault, and, under this suspicion, had cisterns built sufficient for a large supply of rain-water, and filled up the wells. The following year, not a single instance of guinea-worm occurred, and I understand it has never since appeared. In a letter I have lately received from Mr. Templeman, dated Morne Rouge, Grenada, 20th June, 1814, he thus writes to me: (I should have observed, that, on my representation, Mr. Thornton had cisterns built, the happy effect of which is thus shortly stated in this letter:) 'As to the guinea-worm, none have made their appearance in any of the negroes, since about twelve months after your quitting this island; and that twelve months they were more favourable, and did not come to bad ulcers and sores, as you before saw them, nor have I heard or known since that time, now eighteen years, of any guinea-worms being on any of the negroes in this quarter.' "

Several other proofs are produced, after which Dr. Chisholm proceeds.

" All the information communicated by Mr. Templeman, and most of that I received from Mr. Scott, I have often verified on the spot myself. I have examined with precision the well and its water of Point Saline plantation, and the various stadia of the progress of the worm, during the epidemic season. As far as it was possible, by a tolerable microscope, by filtration and other means, I have ascertained the existence, in the water, of extremely minute and agile animalcules, of nearly a cordated figure; and of innumerable white granulated substances, little more than perceptible, even with the magnifier I used, which I concluded, on a comparison of the circumstances I have stated, to be, the former the embryos, the latter the ova, of the dracunculi. Farther than this I found it impossible to proceed in my investigation. More perfect instruments might, no doubt, have produced more perfect evidence of the existence of the dracunculi in the animalcule and egg state.

" Having these facts before me, for the most part minutely observed by myself, and confirmed by the experience of three years, during which nearly 3000 cases of the disease were under my charge, I feel much disposed to offer a speculation on the mode in which the guinea-worm is generated, and received into and evolved in the human body. Whatever can be said on the subject, in the present state of our knowledge of it, must be considered merely as speculative, or can amount to little more than very probable conjecture. Were it possible, indeed, to procure some of the dracunculi in a perfect living state, and to watch their economy in their native medium, then the inferences I am disposed to draw from the imperfect knowledge I have acquired of that economy, might become established facts, and the mystery of their generation, of the mode of their admission into the human body, and of their growth there, might be developed."

Thus we are left in the same uncertainty concerning the origin of these parasytes as of many others whose existence is in the human body. That dracunculi are found in those who bathe in stagnant water in different parts of the tropical world, is well understood; but several authorities are produced, in which they are ascribed to the drinking such water: among others, Mr. Bruce, whose accuracy in all other respects is becoming every day more generally admitted.

The following are the conclusions drawn from the various observations and information made and received by the author.

"On comparing these facts, with those which I myself have witnessed at Grenada, there seems to be sufficient reason to believe, 1. That the dracunculus propagates by ova, or that it is oviparous, viviparous. 2. That the insect affects an argillaceous soil, or one composed of the ferruminated ashes and decayed lava of volcanoes, and more especially when such soil has a considerable impregnation of salt, or when it is percolated by sea-water. 3. That the insect may deposit its ova in this soil, or in the interstices of the skin of the human body, when these are conveniently disposed for their reception. 4. That water percolating through such soil may have the ova floating in it. 5. That, when this water becomes the drink of any part of the human race, the ova are necessarily thereby received into the human stomach. 6. That, therefore, it seems a just inference, that the hatching of these ova may take place in the soil and in the human body. And, 7. That, in the first instance, the insect may in its embryo worm state insinuate itself through the interstices of the skin; in the second may be deposited in its egg state by the secretory organs under the skin, or in the interstices of the muscles, and there hatched."

In the further inquiries, though very minute, we are left in the same uncertainty as before, regarding the mode of generation of dracunculi. The author, however, takes this opportunity of shewing his peculiarly happy prognosis in a case of lumbrici.

"Intestinal lumbrici are certainly oviparous; and there is much reason for believing that they copulate also; for the intussusceptio which proves fatal to young subjects, is occasioned by knots of lumbrici united to each other in the congressional act. I am induced to form this opinion by some instructive cases which occurred to me in Grenada. The first of these took place in the year 1787, of which notes were taken at the time. The subject was a negro child about three years old. To the usual symptoms of intussusceptio, were added most violent spasmodic twitchings of the muscles of all the body, terminating in complete hemiplegia, and inability inability to speak or swallow. Two hours after death, I opened the abdomen, and, as I suspected, found the small intestines filled with lumbrici. But what chiefly engaged my attention and surprise, was to find intussusceptions in seven different parts of the ileum and jejunum; and wherever these were formed, to find them caused by large knots or bundles of worms, which carried the gut from above downwards. In one of these the gut had thus doubled down at least four inches, and the doubled part was considerably thickened and inflamed. My surprise was still more excited on examining the knots or bundles, to find that their union in this state was occasioned by their being copulated or adherent in the manner I had seen earth worms in congressus actu. They did not appear possessed of the acute sense which the earth worms so remarkably exhibit in this state, but permitted themselves to be drawn from each other. Perhaps dracunculi may propagate in the same manner. They are, indeed, much more imperfect animals than lumbrici; but, nevertheless, I cannot accede to the opinion sometimes embraced, that their generation is equivocal, or their propagation effected by offsets or cuttings. My belief is thus expressed by an elegant writer in the Amœnitates Academicæ: "Ne quis tamen inde neget primum in naturæ scientia axioma, quod omne vivum ex ovo, quamvis et Hydra et Gordius in centenas partes divisi renascantur in totidem animalia.' (De Memorabilibus in Insectis, Tom. II. p. 401.) The ova, then, of the dracunculi, being deposited in the water they peculiarly affect, are there hatched at the appointed season."

Many conjectures follow concerning the evolution of these insects in the human body, which we shall pass over with no other remark than that to us they do not appear satisfactory.

Several instances are related of animals whose generation is well understood, whose existence is in the external atmosphere, yet who were found to live in the human stomach and intestines. Of these we see no reason to doubt, as many of the insects and reptile tribe can exist with very little, perhaps, without any, oxygen air, or with no more than they can extract like fish from other substances once supposed to be elementary. Their life would also preserve them from the power of the gastric juice. But nothing of this kind brings us nearer to the knowledge of the origin of those animals which are never formed but in the human body.

Another inquiry follows, how far these dracunculi may be conveyed by contact from one person to another. Here we feel obliged to insert a short passage shewing the extreme candour as well as diligence of this learned writer.

"But, setting aside these disproving facts, if they may be so considered, respecting the contagious nature of dracunculi, it is evident that the "exanthemata viva" can, in no instance, be infectious in the manner supposed to have happened in the 88th regiment.

regiment. 'Insecta ejusmodi minutissima forte acaros diversæ speciei, causas esse diversorum morborum contagiosorum, ab analogia et experientia, hactenus acquisita, facili credimus negotio; neque repugnat horum structura et magnitudo; minima enim sunt animalcula quæ oculus humanus adhuc percipere potuit. Ex sensibus agitur, ad rei impossibilitatem non est argumentandum; æque enim in minimis ac maximis sapientissimi conditoris artificium elucet.' (Amœnit. Academ. Tom. V. p. 94.) In this learned and interesting discussion, the author employs the words contagion and contagious in their literal and legitimate meaning, as they are derived from the verb contingo, and not in the figurative sense in which they are generally applied. By contact, then, colonies of minute insects may emigrate to and form establishments on a healthy person from a diseased one. But such colonizations are confined to insects of the acarus kind; and in all the diseases which the writer of the Exanthemata Viva attributes to the agency of insects, different species of the acarus are alone the cause. The nature of dracunculus is inconsistent with this mode of propagation, more especially under the circumstances the soldiers of the 88th regiment are represented to have been in on shipboard. On the other hand, there is much reason for believing, as I have already said, that the ova of the dracunculi were received into the system by the stomach, and deposited in the cellular membrane, and, having attained their period of evolution, appeared in the persons of the soldiers in such numbers as to induce my respected friend to assign the disease to contagion, in the figurative sense in which the word is usually received. The period of evolution corresponds, it will be observed, precisely with the narrative Sir James Macgrigor has given, and with the history so clearly stated by M. Dubois. The very interesting account of the Acarus siro of Madeira, there called *ouçóes*, by Dr. Adams, is a fine exemplification of the *contagion* of insects, or of the exanthemata viva. (See Medical and Surgical Journal of Edinburgh, Vol. III. p. 343.)"

A short paragraph follows on the prevention and cure of dracunculus. As we had hitherto entertained an opinion, that the only mode of cure was by careful extraction, taking particular care not to break the animal, we shall give this passage entire.

"It now remains to offer my observations on the prevention and cure of the dracunculus. Never was there a disease to which the medical precept, *sublata causa, tollitur morbus*, more distinctly applies than this. The result of the application of this precept at Point Saline, in Grenada, is a manifest exemplification of the means by which this is to be effected, and precludes the necessity for saying more on the subject here. As to the cure of the disease, that is to be accomplished by the destruction of the insect. I used a variety of means, but none effectual, until I had recourse to mercury. Mildly saturating the system with this medicine destroyed stroyed the insect. I since then find that this medicine has been long used by others for this purpose. At that time, 1794, I knew of no authority for it. Linnæus says, 'Infuso mercurii sublimati corrosivi Swietenii intra dies 20, qui alias intra 40 educitur.' (Syst. Nat. Tom. I. p. 2. 1075.) In phthiriasis, and other diseases of the exanthemata viva, mercury has been long known as an effectual remedy externally applied; but in dracunculus it is not so: the remedy must pervade the system, in order to destroy the insect, or its ova. It is unnecessary to detail the variety of means employed in different countries: they are of doubtful effect, however vaunted of—assafœtida, garlic, the root of angelica, sulphur, &c. &c. Some gentlemen, considering the disease as an inanimate substance, recommend the extraction of it by a painful surgical operation; but the opinion is as irrational as the cure is unnecessarily cruel."

We could wish, that Dr. Chisholm had at least hinted something concerning the mode of extraction generally recommended, namely, gradually rolling it round a quill, to which its projecting extremity is fastened. This process we have often heard, though extremely tedious, is the only one which can with safety be relied on.

Art. II.—*On the Relation subsisting between the Time of the Day, and various Functions of the Human Body; and on the Manner in which the pulsations of the Heart and Arteries are affected by Muscular Exertion.* By Robert Knox, M.D. Edinburgh. (Concluded.)

The additions to this paper are unimportant, they, however, are not destitute of use in shewing the necessity of a most minute attention to every thing which relates to action in the living body.

Art. III.—*Observations on the Pathology and Cure of Rheumatism*; by William Balfour, M.D. Edinburgh.

The practical remarks in this paper are so valuable, and the pathological so uncertain and so unnecessary, that we heartily wish the latter had occupied less room. This may appear fastidious to a truly useful essay. But our wish is, that the attention of the young reader should not be led too much astray on a subject which never can be satisfactorily solved; and which, if solved in a manner contrary to Dr. Balfour's opinion, would not lessen the value of his improved practice. That acute rheumatism is an inflammatory disease, cannot be questioned, whether, therefore, it is in the muscles, or the fascia, or the cellular membrane, an antiphlogistic plan is indicated, and, if with this we can gain the assistance of a topical application, our success is more certain and speedy. In the chronic rheumatism, topical applications are particularly desirable, as the constitution is usually in so irritable a state as to forbid free evacuations, and the efficacy of tonics, though often very satisfactory, is also uncertain.

The topical application, recommended by Dr. Balfour, is very tight bandaging. If we may be allowed to offer our opinion on the *modus operandi*, we should say it is by lessening the diameter of the circulating vessels, and thus preventing the means by which local inflammation is supported. That the same may sometimes succeed in acute rheumatism, we have no doubt. It is not, at all times, easy to fix the limits between chronic and acute inflammation; and, as the latter, when not very intense, and the effect of general plethora, will sometimes spontaneously subside, so we are ready to believe, that it may subside more readily by the assistance of topical applications. To shew how ready we are to give the author full credit for his improvement, we think it right to add, that his plan has been adopted in London, and, in some instances, with great advantage.

Art. IV.—*Two Cases of the Dislocation of the Thumb;* by Geo. Ballingall, Esq. Assistant-Surgeon 1st Foot, or Royal Scots. Communicated by John Barclay, M. D. Fellow of the Royal College of Physicians, and Lecturer on Anatomy, Edinburgh.

This is an ingenious paper, and proves in this, as in most other cases of luxation, that more may be done by fortunately seizing a happy moment, than by the greatest violence directed even by the most exact anatomical knowledge.

Art. V.—*Description of a Substitute for Leeches;* by John Welsh, Surgeon, Haddington.

This proposed substitute is a cupping-glass with a tube, through which the air is extracted from the glass, by the common mode of sucking instead of heat or the air-pump. There seems something vulgar in the appearance of such an operation, but it may be convenient, whenever we wish to draw blood from a confined part, or a part not perfectly even in its surface.

Art. VI.—*Case of extensive Wound of the Throat;* by Robert Hathorn, Surgeon, Donaghadee, County of Down, in Ireland.

Art. VII.—*Case of Tetanus cured by Injections of Tobacco Smoke;* by Thomas Duncan, Surgeon, Grenada.

This case, and the reflection upon it, are given with much candour.

candour. The author is very sensible of the uncertainty attending the inference he wishes to draw, but urges with great propriety the necessity of faithful registers in a disease so generally fatal.

Art. VIII.—*Additional Observations on the Treatment of Scrofula;* by WILLIAM GOODLAD, Surgeon, Bury, Lancashire.

In this paper, Mr. Goodlad expatiates on the advantages he has derived from injecting a solution of the sulphate of zinc in the proportion of ℥iſs to 8 oz. or more of water. The use of stimulating applications to indolent sores is now well understood, and washes are generally preferred to greasy applications. We are not prepared to say, whether Mr. Goodlad's formula is entitled to any particular preference. There are, however, many other very judicious observations contained in this paper, chiefly on the necessity of the early opening of such abscesses, and the more free use of the lancet than was at one time adopted; in these, we are ready to admit all the practical advantages mentioned by the author.

Art. IX.—*Notes of the Case of Thomas Watt, whose extensive Wound, and singular Recovery, were the Subject of a Communication from Dr. Haliday.* By JOHN GARRETT, Hospital-Assistant to the Forces.

This is a very interesting case, but not very uncommon in its degree among the numberless incidents which the service presents.

Such are the contents of this Number, which may be truly called respectable. We cannot, however, help regretting, that the critical department appears again degenerating into that severity which we were in hopes had ceased with the death of other periodical attempts. As the medical public shewed its well merited disgust on this occasion, we venture to caution the present editors not to imitate them.

---

*Part of the Introductory Lecture for the Year* 1815, *exhibiting some of Mr. Hunter's Opinions respecting Diseases.* Delivered before the Royal College of Surgeons in London, by JOHN ABERNETHY, F.R.S. Professor of Anatomy and Surgery to the College. pp. from 99 to 136. London, Longman and Co. 1815.

AN advertisement informs us that

"The following sheets comprize only an extract from the introductory lecture of the present year, which is designed to explain some of Mr. Hunter's opinions respecting diseases.

"The

"The pages are numbered, in continuation with those of the introductory lectures of the preceding course, printed last year, in order that the whole may be bound together."

By the above, we should suppose that this makes a sequel of the former lecture, which we felt ourselves obliged to handle with some severity. To the present, we cannot have the same objection, inasmuch as no attempt is made to defend Mr. Hunter's theory concerning life, but only to exhibit his opinions respecting Diseases.

On the subject of Sympathy, Mr. Abernethy says,

"Now, here I would ask, to whom do we owe the first luminous demonstration of this subject; who, first with the eye of a physiologist, surveyed the reciprocal sympathies of the several organs of the body, and shewed how the most complex disorders may and do arise from simple causes? Was it not Mr. Hunter? Allow me, further to enquire, does no good result from this physiological exhibition of the subject? Its utility might be explained by numerous instances, but I shall restrict myself to one. When we see that a compound fracture, in a susceptible and debilitated patient, may so disorder the nervous functions, that all the organs and parts of the body are affected as in that complicated malady a typhus fever; can we longer feel surprized that disorder of the digestive organs and poisonous miasmata should equally and similarly impair and disturb the energies of the nervous system, and occasion this identical fever. Does not this discernment of the causes and nature of diseases, lead to a just appreciation of various remedies, and to judicious practice?

"Whilst considering the constitutional effects resulting from what Mr. Hunter called *universal sympathy*, I further shewed, that the nervous disturbance, induced by local irritation of remote parts, might produce effects more or less general upon the nervous and muscular systems, without so materially affecting the other organs of the body as to engage the attention of common medical observers: and here I spoke of pain, sickness, swooning, rigors, convulsions, delirium, and tetanus.

"Lastly, in considering the effects resulting from these sympathies, I shewed how the nervous disturbance might affect the feelings and functions of the digestive organs, and how the disorder so induced, might, by a reflected operation, augment the former, and greatly and variously disturb the whole system.

"This subject had, indeed, particularly attracted the attention of Mr. Hunter, who believed that the stomach had a direct sympathy with remote organs and parts of the body; whilst he equally observed, how it might reciprocally affect and be affected by the head. It was, on this account, probably, that he was led to call the stomach the centre of sympathies, a term, which such observations, if correct, would render particularly apt and expressive.

"The full importance of this subject could not, I think, have been discovered by the most acute physiological observation. It has has been, however, manifested by the results of medical practice, which shew, that, if the disordered feelings and functions of the digestive organs be removed, the greatest degrees of nervous disorder, will sometimes suddenly cease; at others, will be greatly mitigated, and gradually subside; clearly proving, that, in such cases, the one derangement is the cause of the other."

We believe how much soever Mr. Hunter might consider the stomach as a sympathizer with every other part, Mr. Abernethy was the first to consider every part as sympathizing with the stomach in so extraordinary a manner as to produce so great a variety of local diseases.

The following passage, we confess, appears to us much too allegorical for Mr. Hunter, and even for the subject and the audience before whom it was delivered.

"Lastly, (says Mr. Abernethy,) I spoke of mortification as an event like suppuration peculiar to no individual disease, but a common termination to many. It is the result of simple weakness; of want of action; of excessive action, and of other causes; and, consequently, requiring a proportionate variety of treatment. On this subject, I could not forbear starting an opinion, which some may think whimsical, and others absurd, even before this critical and learned assembly. I asserted, that mortification was not unfrequently the result of nervous disorder in the affected part, and further affirmed, that I was well acquainted with the family from which this disorder, with the subsequent alarming disease, was descended; that it was one of the numerous and dissimilar progeny of the common parents of local diseases; that it was a short lived bantling, engendered by the reciprocal and aggravating irritation of disordered states of the digestive organs and nervous system on each other. It therefore followed, that local applications were of little avail in this species of mortification, and that the removal of the exciting causes were the chief means of procuring a diminution and ultimate cessation of such effects."

That local applications to a dead part can be of little avail will hardly be questioned, nor can we doubt, that in the subsequent lectures all the figures of the above quotation are explained; but we are not quite reconciled to meeting with any thing in print which is not explained by the same medium.

Most of all, we wish Mr. Abernethy would take a lesson from his master, whom he extols, we doubt not, with equal truth and gratitude.

"To me the philosophical turn of Mr. Hunter's mind is demonstrated by his caution; with all his facts relating to sympathy, he formed scarcely any general conclusions. He distinguishes it into the continuous, contiguous, and remote. The two former are readily explicable. Of the latter, I, who have less caution, or more facts of a certain description than Mr. Hunter might have possessed, do not hesitate to say, that when injuries or disease of limbs, bring on fevers, delirium, convulsions, or tetanus, or disturb the feeling and functions of the digestive organs, that these effects are produced through the medium of the brain."

After a few other remarks, the same paragraph concludes.

"To me it is evident, as it was to Mr. Hunter, that the stomach has a direct sympathy with the most distant parts of the body; and that the heart sympathizes with the stomach; but, in what manner such sympathies are produced, or how the morbid and irregular sympathies which occur in diseases are occasioned, we presume not to explain. Yet, if Mr. Hunter's opinions of the nature of life be true, none of these facts can well be considered as surprising."

This may be all very plain to those who understand Mr. Hunter's opinions of the nature of life; but such is not the case with us.

In the former part of this paper, Mr. A. had hinted to us his distinction between disease and disorder; here he explains his meaning:

"Disorder, (says he,) which is the effect of faulty actions of nerves, induces disease, which is the consequence of faulty actions of vessels."

We have now given enough to teach the reader the general form of this lecture. If we understood it better, we would offer our opinion of its merits; but, either from our own dullness, or for want of hearing the rest of the course, we must acknowledge that to us it is obscure. While, therefore, we are ready to admit the just claims of Mr. Abernethy to the rank he holds in the scientific world, we cannot help expressing our wish, that he would prevail on some impartial friend to peruse his manuscripts, and not send them to the press till he finds that friend admits them to be as perspicuous as they are doubtless ingenious.

---

*Letters addressed to his Royal Highness the Duke of Kent, on Consumption; containing Remarks on the Efficacy of Equable and Artificial Temperature in the Treatment of that Disease, &c.* By Thomas Sutton, M.D. of the Royal College of Physicians, late Physician to the Forces, and Consulting Physician to the Kent Dispensary; and Author of Tracts on Delirium Tremens, Gout, &c. 8vo. pp. 59. London, Underwood, 1814.

Our neglect in not noticing this treatise sooner, is accidental. The interest of the subject, however, though probably of a passing nature, is not yet gone by. The institution, against against which the present pamphlet is directed, not only exists, but seems to be gaining popularity. In a disease so extensive and so fatal, so insidious in its access, and deceitful in its progress, as is pulmonary consumption, we cannot be surprised that every new attempt to cure it is hailed with avidity.

As we presume the Infirmary for the Relief of Consumptive Disorders, will admit them at any period or stage of progress, we shall confine our observations to the commencement of those disorders; for, if seclusion within heated apartments will not cure phthisis, at least we know of no harm that can arise from it. The question to be decided is, how far such an institution, where patients are confined in an artificially heated atmosphere, at a temperature of 65°, is likely to be of public benefit in the cure of pulmonary complaints? Facts will support the practice, unquestionably, in some instances. But the warmest advocates of the measure well know, that, in private practice, with every comfort that wealth could procure, or affection demand, it has failed in almost every instance of pulmonary consumption. If it has succeeded in the early stage of that disease, we also know that very opposite means have proved yet more successful, such as a sea voyage and horse exercise; and we have heard of cases, where even the application of cold to the chest, on the principle that there was local inflammation, was of service. It is also a well-attested fact, that, in general, individuals who shut themselves up in warm apartments, where the external air is cautiously excluded, are more susceptible of catarrh and cough, than those persons who are constantly exposed to the air, and whose houses are more permeable. Considering then the class of patients who are likely to become the inmates of an infirmary, and the facility with which they are relieved at dispensaries, not appropriated to any particular disease, but where all complaints are also treated, we have little hesitation in determining, that the proposed institution is unnecessary, and that the treatment intended to be pursued is objectionable. We say objectionable, because we cannot deny, that patients labouring under cough may not be cured by the means proposed; but we affirm, that they would be unfit, in many instances, during the winter, to pursue their usual occupations. Would, for instance, a woman who, to gain her daily bread, has no resource but sitting by a barrow in the open air from morning till night, or from night till morning, be fit to be turned out of a snug chamber, heated to 65°, any time between November and March. This is not an extreme case.

Such

Such were our sentiments before we took up Dr. Sutton's treatise, which we find is directly opposed to the shutting-up system.

"This institution (he remarks) is not proposed to be founded upon principles of common humanity, by which we are led to raise an institution for the reception and recovery of the poor; but upon a particular principle, that diseases of the lungs, in the foremost ground of which are consumptions, require a peculiar mode of treatment, and constructions adapted for their peculiar nature; and the prominent object is to place persons subject to these diseases, and to retain them in an artificial or factitious atmosphere, always equal to the temperature expressed by Fahrenheit's scale of 65 degrees; that is, the medium summer heat of this climate."

To this, Dr. Sutton objects that the plan has been sufficiently tried, but without success; on the contrary, he infers, from his observations, that "the patients who have submitted to this regulated temperature, or an attempt at it, have recovered in less proportion, and been the soonest precipitated to the grave."

If we go to Madeira, to Sicily, or to Malta, we find consumption prevail amongst the natives to a considerable degree, and few strangers return to boast of their cures. Large experience has long since demonstrated the inefficacy of confinement in heated apartments in confirmed phthisis, which, indeed, is rendered sometimes more insupportable by its tendency to increase the fever.

"It must be evident (the author continues) that heated rooms cannot concur to health and vigour; invariable in temperature, confined in circulation, unpleasant to the parties inhabiting them; and who, on this account, are rendered listless, and by no means desirous of the least exercise, which would soon tend to an increase of heat highly uncomfortable to the feelings. But if these objections exist to such rooms set apart for equable temperature, there is another consideration worthy of attention, which is, that not only the temperature possesses the objections I have stated, but the air is invariably dry: and that this is important to be considered, must be clear from the circumstance, that those who respire the most dry air are the most subject to pulmonary consumption. This fact is evident, from the disease occurring most among the higher classes in this country, and more particularly among the females in that class; also among those whose sedentary occupations allow them to be very little in the external air; also among bakers, whose occupations lead them to respire the dry air of their bake-houses; and manufacturers, whose employments are connected with fires. It is true, that there are those who suffer from the disease under all circumstances; but the greater number by far will be found to be be those who, from various circumstances, are placed in situations to respire a dry atmosphere.*

"Some observations made to prove, that, where intermittents prevail, consumption is less common; and the contrary tend also to elucidate this statement. The world is indebted to Dr. Wells for investigating these facts; who, on this and other subjects, has given much excellent information.† The intermittent, as is known, prevails in marshy situations, where the atmosphere must be constantly loaded with a considerable degree of moisture. It must be evident, that the constant return of this disease tends to render the inhabitants of such situations very unhealthy; but they suffer comparatively less from consumption than their neighbours who inhabit drier situations. One would at first imagine, that intermittents would promote the prevalence of consumption, as we find it in other situations frequently to follow every species of disorder that tends to lower the habit. But though marshy districts do supply a multitude of disease, consumption is not one of them, contrary to what happens under different circumstances. As, therefore, we find unhealthiness in other situations not to counteract consumption, we can hardly, with any species of propriety, attribute this greater freedom from that disorder to the effects of the agues, but ought to conclude that it is caused by something common in the one country, and which is not equally possessed by the other; and this, it may readily be concluded, to be the moisture of the atmosphere.

"Another circumstance that has fallen under my observation, which has tended to strengthen this opinion in my mind, is, that consumptive patients appear to derive advantage in the winter

---

* "Consumptions in this country have been almost uniformly considered to be caused by the variableness of our climate, and humid atmosphere; and we have been supposed to be more afflicted with this disorder than other parts of the world not so affected. Hence have arisen the efforts to cause an artificial climate of great uniformity. But it must be suspected that there is error in these suppositions, since we learn that consumptions prevail much even in the most favoured regions as to temperature and dryness. We undoubtedly find inflammatory affections to abound with us in very variable weather, and inflammations of the lungs among the rest, but these latter more frequently tend to excite into activity the consumptive disposition already existing, than become the real causes and grounds of the disease; for, when this disposition does not prevail, ulcerations of the lungs very frequently heal both kindly and rapidly. There can also be no doubt, that with this disposition a considerable proneness to inflammation in these organs exists, which will cause the disease often to commence in such seasons, and most frequently to be wholly attributed to them.

† Vide Medico-Chirurgical Transactions.

time time from some parts of Devonshire, such as Sidmouth, &c. which is a country, as I am informed, subject to great fogs at that time of the year. Several instances have occurred in my connexion, of such patients passing their time very beneficially in these situations. Something of the same kind also appears to have been observed by the justly celebrated Dr. Cullen. 'In his lectures on this disease (consumption), he was wont to observe, in directing a change of climate,' (says Dr. Gourlay,) 'that it was as pernicious for phthisical patients to pass the summer in a very warm climate, such as Madeira, as to remain in England in winter; and, indeed, that the most benign climate, in such cases, was found in the south of England, and in the winter of southern latitudes.* In fact, it is probable that Dr. Cullen had more opportunities of noticing the effects of the climate of the south of England on consumption, than of any other southern climate; and though he might not advert to the circumstances I have alluded to above, yet his testimony may be in favour, perhaps, of the very places I have hinted at, because they are much resorted to by the consumptive, who are led to take up a residence in that part of the world. It is not improbable also, that voyages by sea produce their beneficial effects on the disease, through the moisture of the air. Their utility to the consumptive has very long been held in estimation: and among the Romans this remedy was recommended, with the object of profiting by the advantage of a denser atmosphere.

As we believe the able and candid physician who mainly supports, and is at the head of the institution for consumption, &c. is perfectly sincere in his conviction of its efficacy; we hope, that, should he object to any thing that is here urged against the plan, he will favour us with his remarks, which we shall not fail to communicate to our readers; for public good is our sole object in offering our opinion on the present occasion. If in error, we are willing to be convinced.

---

*The Morbid Anatomy of the Liver; being an Inquiry into the Anatomical Character, Symptoms, and Treatment, of certain Diseases which impair or destroy the Structure of that Viscus.* Order I. Tumours. *Part* II. *On the Varieties of the Tubera Diffusa.* By J. R. Farre, M.D. Imperial 4to. two plates. Longman and Co. 1815.

In our Number for February, 1813, we noticed the first fasciculus of this splendid work, which contains an account of the tubera circumscripta, and the tubera diffusa. The second part, which, after an interval of about three years, has

* Vide Gourlay on Madeira, and London Medical and Physical Journal, vol. v. p. 307.

has just come out, continues the subject of tubera diffusa, and begins with an account of the varieties. These are so numerous, that Dr. Farre declines describing all that he has met with, and contents himself with illustrating, by cases, some of the most remarkable.

The first variety, of which he gives an example, is

"II. 1. CHARACTER. Tubera, affecting different textures, viz. those of the liver and stomach;—elevated at the surfaces of the organs, *but not uniform in their figure, some rising with a regular swell into a round form, others acquiring a margin, by being gradually depressed towards the centre*;—forming tumours without cysts, almost pulpy in their consistence, cellular in their structure, and containing an opaque white fluid."

Of the symptoms the author says very little; the following short paragraph contains all the information he is pleased to communicate on the subject.

"SYMPTOMS. As the anatomical characters of both species are combined in this variety, so the symptoms are proportioned to the growth of the tumours in their respective organs. *With the addition of those signs which result from the affection of the stomach*, the symptoms attached to the character of the tubera circumscripta will not only distinguish the genus, but also the species."

A case of this disease is stated.

The second species is thus described:—

"II. 2. CHARACTER. Tubera affecting different textures, elevated at the surfaces of the affected organs, *encysted, or having distinct cells,* formed by the growth of a fungus, which separates in flakes, and is composed of a fine reticular texture, containing an opaque white fluid."

As the symptoms of this variety are not enumerated, we shall quote a case of it, which the author received from a friend, together with a recent specimen of the tuber.

"Mrs. W. æt. 48, spare and diminutive in her figure, and temperate in her habits, had complained, for the preceding two or three years, of pain in the epigastric region; and, during the last twelve months, although her bowels continued regular, she was affected with frequent vomiting. Within the last three months, ascites and slight symptoms of jaundice had taken place; but for a month previous to her death, the yellowish state of her skin and tunica conjunctiva had disappeared. She said, that every medicine which she took, served only to increase the pain of her stomach. The continual suffering of this organ was expressed by her constant employment of gently rubbing the epigastric region with her hand. Her respiration was little affected; her pulse was small and quick; and her debility great."

"DISSECTION. The liver was not remarkably enlarged, which it almost uniformly is in these cases, but its external surface was irregular, from numerous tubera, and its internal substance was filled with those tumours. Some of the tubera had distinct cells, or even cysts, which contained a white substance, of the consistence of cheese, capable of being separated in flakes. From the surfaces of the tubera, after a fresh section, a white fluid, as thick as cream, could be scraped. A portion of the tumour, after many days' maceration, presented as fine reticular texture as the structure of the tubera, in which that fluid was contained. The mucous coat of the stomach, near the pylorus, had an irregular tuber growing from it. The peritoneum contained about six pints of serum. In the thorax, the only morbid appearance was a general adhesion of the pleura of the left lung to the pleura costalis.—

The character of the third species, as described by Dr. Farre, II. 3, is

"Tumours, rising with a regular swell from the surfaces of the affected parts, and *yielding to the touch;* composed of a very delicate reticular texture, *pulpy in its consistence*, varying in its colour, even in the same subject, charged with an opaque fluid, and *growing from cysts, or cells.*"

"Symptoms. This variety can be distinguished from the preceding varieties of Tubera, only by the pulpy consistence of the tumours, which impart a sensation resembling, in some degree, what is experienced when a swelling, that contains a deep-seated fluid, is touched."

The tumours affecting the liver are secondary; the primary ones commencing in distant organs, or in the cellular membrane. The disease attacks children as often as adults, whilst the preceding varieties are found in the middle or advanced periods of life.

The author also remarks, that, whilst no texture, cartilage perhaps excepted, escapes its ravages, it most frequently commences in the internal tunics of the eye, in the female breast, and in the testicle. Some cases of the disease are stated, and an elegant plate illustrates the appearance of this variety.

The character of the fourth variety, which concludes the present fasciculus, is,

"Tumours elevated at the surfaces of the liver, and inclining to a round figure; pulpy in their consistence, being charged with a thick and opaque fluid; variegated in their colour, chiefly white mingled with red, the former prevailing in their incipient, the latter in their advanced stages; composed of a *very vascular* and reticular texture, attached either to distinct pouches, or to the substance of the liver; and so unlimited and rapid in its growth, as to burst or destroy the peritoneal tunic of this organ, and to protrude in the form of a bleeding fungus."

This variety is so like the third, that it is not to be distinguished from it by the symptoms during life.

The following is the only case of the disease recorded by the author.

"W. D. æt. 41, by occupation a mechanist in a brewhouse, a man of large stature, vigorous and athletic, given to the intemperate perate use of malt and spiritous liquors, came under the care of a surgeon, for the cure of an ulcerated leg, in the spring of 1812. This gentleman did not, at that time, remark any derangement of his general health; but was informed that his stomach had been disordered, about two years previous to that period, to an extent which had induced him to consult a physician for the complaint. On the 5th of November, 1812, he again consulted his surgeon. His general appearance then indicated visceral disease. His pain was chiefly seated in the left hip and left iliac region; it was sufficient to interrupt his sleep, and was even attended with some degree of lameness when he was exposed to cold. He still, however, continued his employment in the brewhouse. Three grains of the submuriate of mercury, with twelve grains of the compound powder of ipecacuanha, and five grains of rhubarb, were prescribed for him at night. On the 7th, the pain of his hip had abated; but he complained of nausea and pain of the stomach. The submuriate of mercury was given in combination with the compound extract of colocynth, and was assisted in its operation by doses of the sulphate of magnesia. On the 10th, he complained of uneasy feelings in the epigastric region, and his surgeon, by a careful examination, ascertained that the liver was not only enlarged, but presented a tuberous surface. The former medicines were changed for the mercurial pill and the extract of conium. On the 13th, a blister was applied over the epigastrium; but as the prevailing pain still extended from the left hypochondriac region to the left hip, he was cupped on that side. On the 21st, the extract of conium was continued; he also took a grain and a half of opium and six grains of the antimonial powder at night, with much relief. Opium always had this effect until the disease passed into its last stage. From the 22d of November he was committed to the care of a physician, who, on his first visits, made the following observations. He was extremely feeble, his appetite was bad, and he had considerable uneasiness at his stomach, even after a very moderate repast. He complained of great pain in the abdomen, above the umbilicus, but most particularly in the epigastrium and in the region of the liver. These parts, when examined, were tense and painful. As the disease advanced, the enlargement of the liver, affecting both lobes, became very susceptible through the parieties of the abdomen, extending nearly to the umbilicus; and considerable inequalities could be distinctly traced upon its surface. As the body became more emaciated, this inequality of the surface, as well as the enlargement which accompanied it, became still more distinct. Pressure produced more pain when made on some parts of the organ than on others. His countenance was remarkably sallow; but there was no appearance of jaundice, and the albuginea of the eye was of a pearly white. His urine was turbid, and deposited a copious sediment on cooling. Although his stools were variable in colour and consistence, yet they did not afford those appearances which are indicative of a very deficient biliary secretion.

His pulse was at this time about 80; but towards the close of his life it varied in frequency from 100 to 112. He was treated by a grain of calomel and a grain of opium every night, and occasionally by aperients. His sufferings, however, were too severe to be much alleviated by medicine; and finally, even opium, in moderately large doses, procured him but little sleep. The dyspnœa was inconsiderable, and, in every stage of the disease, he was free from cough. About a week before his death, his food began to be rejected by vomiting, and he was frequently troubled with singultus. At this time, an effusion into the abdomen was very perceptible, his strength rapidly declined, and he died on the 27th of December, 1812. His body was examined on the 30th of the same month.

"External Appearances. The abdomen, owing to the fluid effused into the peritoneum, was uniformly swollen; but, when the epigastric region was pressed, the enlarged liver was distinctly felt, and two tubera could be traced on its surface.

"The rest of the body was emaciated.

"Abdomen. On dividing the parieties, many quarts of serum, deeply coloured with red particles, escaped, and numerous filaments of crassamentum were found adhering to the intestines. A considerable portion of the liver appeared below the hypochondria, and occupied the whole of the epigastric and a part of the umbilical regions. Numerous tubera were seen on the convex and concave surfaces of both lobes. They had a motley appearance, and rose with a gradual swell from the liver. Their contents were pulpy, of a whitish colour mingled with red, and, when compressed, a cream-like fluid, tinged with blood, rose in abundance from their reticular texture. In two distinct parts of the eighth lobe, its peritoneal tunic had been ruptured by soft fungi of a red colour, from which the sanies had issued. Some of the tubera were distinct, the fungous structure being attached to cells, and the intervening portions retaining the character of the liver; but many of the tumours coalesced, and the boundaries between the natural and morbid structures were indistinct. The gall-bladder contained a very little bile of a bad quality. The parts about Glisson's capsule were so united into one mass, as to prevent any dissection of the biliary ducts and vessels. The stomach, being thrust much out of its natural position by the enlarged liver, and by the clusters of tubera which occupied its lesser curvature, and surrounded its pyloric extremity, appeared unusually low on the left side. Its tubera were still more pulpy than those of the liver; and their fungous structure, of a very red colour, was either contained in a large cyst, or grew from numerous small, and very distinct pouches. The mucous coat of this organ was much thickened, and its surface was irregular. Here and there a small cyst appeared in it, filled with the fungous structure. This coat was so much thickened as to narrow the pylorus, and so pulpy on its surface at that extremity, as to present an appearance resembling ulceration. Numerous lymphatic glands in the course course of the aorta were converted into tubera. The rest of the abdominal, and all the thoracic, viscera were sound."

We have now stated the chief contents of the present number, and have been copious in our extracts, both on account of their merit, and because the expence of the work will probably confine it to a very limited number of readers. As far as we can judge from the two numbers which we have seen, the work, when completed, will possess a very strong claim to professional notice; no one, indeed, can peruse the specimens which we have quoted, without feeling the advantage of becoming more intimately acquainted with the diseases of an organ, which, though so frequent, appear to be so little understood.

---

*A Treatise on the Art of Cupping, in which the History of that Operation is traced; the various Diseases in which it is useful indicated, and the most approved Method of performing it described.* By Tho. Mapleson, Cupper to his Royal Highness the Prince Regent, to the Westminster Hospital, and the St. Pancras Parochial Infirmary. 12mo. pp. 80. Callow. London.

Mr. Mapleson, it is well known, has long enjoyed an extensive practice in cupping. We were, therefore, prepared to expect some useful observations from his pen, and we have not been disappointed. Some individuals, indeed, have objected to his pointing out the diseases in which the operation is more especially beneficial, on the principle of his not being a practitioner of medicine. We see no great force in this objection, and, judging the work by its merits, can safely recommend it to young practitioners. The historical sketch of the operations of cupping, as related by the author, contains some very curious information; and the practical instructions for performing the operation itself will be perused with great interest by all who may ever be in the way of operating, which, in country-practice, is not unusual; though it is often performed in a bungling and slovenly manner. We shall conclude with transcribing Mr. Mapleson's mode of cupping.

"When about to perform the operation, let there be provided a hand-bason with warm water, a piece of fine sponge, and a lighted candle. Place as many glasses in the bason as may be judged requisite to draw the quantity of blood intended to be taken away. If sixteen or twenty ounces are ordered, four glasses of a size adapted to the surface, will in most cases be required. Each glass is then separately to be held, for *an instant*, over the flame of the burning lamp, and immediately placed upon the skin of the patient. Upon the quickness with which this is done, depends pends the whole neatness and efficacy of the operation.—To obviate their want of dexterity, many operators in the country throw a little bit of tow, dipped in spirits, and inflamed, (ignited) into the cupping glass, the moment before it is applied, a very clumsy expedient, adding unnecessarily to the sufferings of the patient by cauterizing the skin ; doing harm also by rarefying the air more than necessary within the glass, in consequence of which the edges of the cup compress the cutaneous vessels so much as to obstruct the influx of the blood.

" If the glasses have been duly exhausted, the skin will be seen gradually to swell up within the cup, owing to the pressure of the air upon the parts in the vicinity, as well as the expansion of the fluids contained in the cellular membrane. The skin becomes also of a dark purple colour, owing to the influx of blood into the smaller vessels. If dry cupping be only intended, the glasses may be allowed to remain on the skin for a few moments, and replaced five or six times, varying their position a little, to prevent bruising the skin.—If the intention be to scarify and take away blood, the glass ought not to remain more than a minute, when it is to be removed by gently introducing the nail of the fore finger under the edge, and the scarificator *instantly* applied and discharged upon the skin, before the tumour has had time to subside. Upon the rapidity or slowness with which the application of the scarificator succeeds the removal of the glass depends all the sufferings of the patient. If the skin has completely subsided before the stroke of the instrument, much unnecessary pain is inflicted.

" The glasses are then to be removed and re-applied in succession. They should be a second time removed, if necessary, as soon as the blood is perceived to coagulate within them, or when they are so full as to be in danger of dropping off. For the sake of neatness, care should be taken to insert the nail under the upper part of the glass, and open them downwards, gently wiping the wounds at the same time with a warm sponge."

Some further useful remarks follow, but we have already stated enough to shew the manner of proceeding, and to prove the advantages of performing the operation neatly and expeditiously.

---

*A few Hints relative to Cutaneous Complaints;* by T. M. Kelson, Seven Oaks. 8vo. pp. 20. 1814.

A question may possibly arise in the minds of the readers of this pamphlet, under what description the author should be announced? His own candid explanation, however, we think will enable them to determine rightly on the occasion. He presumes that he possesses a remedy for the cure of pimpled faces, which he does not choose to divulge, it not being convenient, as he alleges, at present to allow his brother brother practitioners to participate in his emoluments. But the preface, of which the following extract constitutes the better half, is intended to exonerate him from any implication of quackery.

"To pretend to be exclusively in possession of specific means of cure, and to withhold those means from the profession, I must acknowledge, carries with it rather an air of quackery; but I flatter myself, my immediate medical and other friends will give me credit for not having a particle of that sort of spirit in my composition."

Of course, no information can be gained from such a work.

